# A New Way to Detect Stone in the Bladder

**Published:** 1882-09

**Authors:** 


### A New Way to Detect Stone in the Bladder.

Dr. James McKenzie Davidson, in the *Lancet*, calls attention to the "auditory method" of detecting vesical calculi. This simple method consists in connecting the end of the catheter with the ear of the surgeon by means of an India rubber tube. A light rubber tube two feet long and with a bore three-eighths of an inch in diameter was employed. One end was slipped over the handle of the sound and the other end held close to the ear. No difficulty was experienced in exploring the bladder, because the tube was so flexible, and it only required a little care to prevent the interference of extraneous noises, as rubbing against the coat, etc. A small calculus was introduced into the bladder (for experiment) and when nothing could be heard or felt by the sound alone, yet by means of the tube the calculus was distinctly and unmistakably heard. With a large stone the click was greatly intensified when heard through the tube. A small piece of coal was crushed to rough powder and introduced into the bladder. The ordinary method revealed nothing, but through the tube a rough, grating sound was distinctly heard.

This method of exploration of the bladder may yield important practical results. Not only may (1) a small calculus be detected which would be otherwise overlooked, but (2) it may be that practice will enable the operator to distinguish the size and character of the surface of a calculus readily; and (3) it also appears likely that a somewhat similar ear connection with a lithotrite will enable the operator to find and secure small fragments more rapidly, and so crush them.

